# The Carter Center Mental Health Program: Addressing the Public Health Crisis in the Field of Mental Health Through Policy Change and Stigma Reduction

**Published:** 2006-03-15

**Authors:** Rebecca G Palpant, Rachael Steimnitz, Thomas H Bornemann, Katie Hawkins

**Affiliations:** The Carter Center Mental Health Program; The New School, New York, NY; The Carter Center Mental Health Program, Atlanta, Ga; Georgia State University, Atlanta, Ga

## Abstract

Some of the most pervasive and debilitating illnesses are mental illnesses, according to World Health Organization's *The World Health Report 2001 — Mental Health: New Understanding, New Hope*. Neuropsychiatric conditions account for four of the top five leading causes of years of life lived with disability in people aged 15 to 44 in the Western world. Many barriers prevent people with mental illnesses from seeking care, such as prohibitive costs, lack of insurance, and the stigma and discrimination associated with mental illnesses. The Carter Center Mental Health Program, established in 1991, focuses on mental health policy issues within the United States and internationally. This article examines the public health crisis in the field of mental health and focuses on The Carter Center Mental Health Program's initiatives, which work to increase public knowledge of and decrease the stigma associated with mental illnesses through their four strategic goals: reducing stigma and discrimination against people with mental illnesses; achieving equity of mental health care comparable with other health services; advancing early promotion, prevention, and early intervention services for children and their families; and increasing public awareness about mental illnesses and mental health issues.

## A Public Health Crisis

Some of the most pervasive and debilitating illnesses are mental illnesses. According to World Health Organization's (WHO's) *The World Health Report 2001 — Mental Health: New Understanding, New Hope*, approximately 450 million people worldwide have neuropsychiatric conditions; those conditions account for four of the top five leading causes of years of life lived with disability in people aged 15 to 44 in the Western world (1). Furthermore, Global Burden of Disease 2000 in Aging Populations estimates indicate that mental and neurological conditions account for 30.8% of all years lived with disability (1). Some of the more common mental conditions are depression, dysthamia, bipolar disorder, schizophrenia, and anxiety disorders. Depression causes the most disability, accounting for almost 12% of all disability (1).

Despite these statistics, mental health and mental illnesses remain issues of which the public is largely unaware. Many individuals with mental illnesses are not diagnosed or do not receive treatment for their illness. Annually, more than 26% of the U.S. population will be diagnosed with a mental disorder (2), and only about one third (8.6%) of those will receive treatment (3). If left untreated, these illnesses often have dire consequences. In fact, in 2002, suicide was the leading cause worldwide of intentional violent deaths (Figure), surpassing both homicide and war (4).

**Figure:**
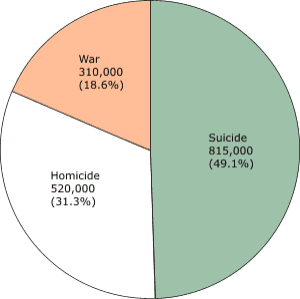
Number and percentages of violence-related deaths worldwide, according to World Health Organization's *World Report on Violence and Health*, 2002 (4).

The cost of untreated mental illness is staggering and has a significant impact on the U.S. economy. A study conducted on the effects of depression in the workplace found that depression caused a loss of $44 billion each year in both presenteeism (the act of remaining on the job but not being as productive because of illness or stress) and absenteeism. This figure was approximately $31 billion more than the amount lost for nondepressed workers (5).

In 1999, Surgeon General David Satcher released *Mental Health: A Report of the Surgeon General* (2), which focused on mental illnesses and mental health issues in the United States and issued a call for action in the field of mental health care. According to the Surgeon General, the financial cost of mental illness to the nation is $63 billion in lost productivity because of illness and $12 billion because of premature death (2). The financial burden placed by mental illnesses on the United States points to the need for a concerted effort to focus on prevention and early identification of those illnesses.

Depression and mental disorders are also highly comorbid with other serious medical conditions: nearly 15% of individuals exhibiting a mental disorder in 1 year have a co-occurring disorder, compared with 3% of the general population (2). Major depression is correlated highly with diseases such as diabetes, coronary artery disease, chronic arthritis, and stroke (6). Smoking also is strongly correlated with depression. In any given month, people with mental illnesses (representing 28.3% of the U.S. population) consume approximately 44.3% of cigarettes smoked nationwide, based on a nationally representative sample, and people with mental illnesses are nearly twice as likely as individuals without mental illnesses to smoke (7). Treating an individual with multiple comorbid disorders can be costly, and it can be challenging to coordinate care among diverse medical providers.

Compounding those existing challenges, the age of onset for many major mental illnesses is young. Kessler et al (8) reported the following age-of-onset interquartile ranges: 8 years (age 7 to 15 years) for impulse-control disorders and anxiety disorders and 9 years (age 18 to 27 years) for substance use disorders. Based on their findings, Kessler et al noted that these patterns are opposite of those associated with chronic physical disorders, and risk increases into middle and late age. Prevention and early intervention of mental illnesses are crucial because they appear to have the strongest foothold during youth. By identifying and aggressively treating mental illnesses, particularly in children, disability could be lessened or prevented, thereby reducing the financial burden to society. 

Finding and obtaining effective medical treatment for mental illnesses, however, can be extremely difficult, particularly for racial and ethnic minorities. The disparities in care for racial and ethnic minority groups pose a significant public health crisis because racial minorities are continually underserved by mental health providers. Only 16% of African Americans with a diagnosable mood disorder seek care from a mental health specialist, and only approximately 29% seek care from any medical provider (9). African Americans and Latinos are substantially less likely to use some form of depression treatment than whites (9). For example, 70% of all children and adolescents who need treatment do not receive mental health services (2), but African American and Latino children have the lowest rates of service use, even when controlling for insurance status (10). If these children are left untreated, the persistence of these disorders may eventually lead to school failure, poor employment opportunities, and poverty in adulthood (11).

Many barriers keep people from seeking and obtaining care. Many racial and ethnic minorities live below the poverty line or do not have health insurance. Nearly one in four African Americans is uninsured (9), and few mental health care providers are minorities. For every 100,000 Hispanic individuals in the United States, there are only 29 Hispanic mental health care professionals (9). These two barriers are compounded by the stigma surrounding mental health issues in both minority and nonminority communities. In addition, some cultural and familial values do not support help-seeking behaviors; thus, many racial and ethnic minorities remain without care.

## The Carter Center Mental Health Program

Former First Lady Rosalynn Carter has been a driving force in the field of mental health for more than 30 years, educating policy makers and the public about mental illnesses. During the Carter administration, she served as active honorary chairperson of the President's Commission on Mental Health, the first presidentially mandated commission examining the mental health needs and policies of the United States. Upon returning to their home state of Georgia after the presidency, President and Mrs Carter formed The Carter Center, an internationally recognized nongovernmental organization with the goal of advocating for human rights and alleviating human suffering. In her role as chairperson, Rosalynn Carter developed in 1991 The Carter Center Mental Health Task Force, consisting of a diverse group of professionals such as mental health experts, advocates, medical professionals, and two former Surgeons General. These professionals oversee The Carter Center's mental health initiatives and spearhead the annual Rosalynn Carter Symposium on Mental Health Policy.

The Carter Center Mental Health Program supports the implementation of policy change with the Mental Health Task Force, whose purpose is to identify major issues concerning mental health and mental illnesses and develop initiatives to reduce stigma and discrimination. The Carter Center Mental Health Program's approach to mental health policy is broad and includes activities to promote mental health, prevent mental illnesses, and support treatment through mental health services. Through its action-oriented agenda, The Carter Center Mental Health Program supports policies and services that better the lives of people with mental illnesses and their families. The goals of The Carter Center Mental Health Program are consistent with the principles of The Carter Center, which emphasize action, results, and the belief that people can improve their lives and the lives of others when provided with the necessary skills, knowledge, and access to resources.

### Program projects

**The Rosalynn Carter Symposium on Mental Health Policy**

The Rosalynn Carter Symposium on Mental Health Policy was established in 1985 and brings together a group of mental health care professionals, including government agencies, consumer groups, and advocacy organizations. Each year, the symposium focuses on a different mental health policy issue. Past topics have included ensuring quality mental health care, promoting healthy behavior in children, and mental health and illnesses in the workplace.

The Carter Center has a nonpartisan philosophy and works to convene individuals and organizations for open discussion and provide people with access to the highest levels of knowledge in the field of mental health. The 2003 symposium dealt with implementing the recommendations from the final report of the President's New Freedom Commission on Mental Health, *Achieving the Promise: Transforming Mental Health Care in America* (11). The keynote address was given by Michael Hogan, chair of the commission. Three panels examined and discussed the implications of mental health science for society, moving sciences to services, and strategic implementation of the goals in the final report.

Symposium participants were affiliated with local, state, and national government agencies, professional organizations, nonprofit organizations, family and consumer advocacy groups, service providers, research and educational institutions, and the media. After the event, The Carter Center conducted a survey to gain insight into the participants' perceptions of the impact of the commission, and the action their organization took based on commitments expressed in work groups at the symposium. The survey's methodology has been published elsewhere (12). Forty-one percent of 68 respondents believed that their organization's behavior changed in response to the commission; in addition, many felt that the report encouraged partnerships with external organizations in working toward goals of policy change and mental health (12).

**Rosalynn Carter Georgia Mental Health Forum**

The Carter Center Mental Health Program also hosts the Rosalynn Carter Georgia Mental Health Forum. Established in 1995, the forum convenes state mental health officials and has focused on various topics, including "Children's Mental Health and Recovery: A Journey for Life." The most recent topic, "Moving Medicaid to Managed Care in Georgia," focused on the privatization of Medicaid in Georgia and provided an opportunity for policy makers from areas such as government agencies and advocacy groups to convene and discuss the implications and the possible consequences of the new policy.

**Conversations at the Carter Center**

The center's four-program series, Conversations at the Carter Center, is a public outreach program that has been used at strategic points to educate the public about mental health issues. The format includes expert speakers about topics related to The Carter Center's program activities with audience participation through questions and answers. Previous topics have included, "Coping with the Stigma of Mental Illness," "Breaking Through the Stigma: Portrayal of Mental Illness in the Media," and "Navigating the Children's Mental Health System." 

### The Rosalynn Carter Fellowships for Mental Health Journalism

The Rosalynn Carter Fellowships for Mental Health Journalism were established in response to the knowledge that the public often bases its perceptions about people with mental illnesses on the media's depictions of them. The single greatest barrier preventing people from accessing mental health care is stigma (11). According to the National Mental Health Association, the media is largely responsible for the persistence of misconceptions about mental illnesses (13). A 1997 study for the Health Education Authority in the United Kingdom found that newspaper stories linking mental illnesses with violence and crime were more prominent than positive stories about mental health. The study also found that almost half of the media coverage of mental illnesses was presented negatively and usually associated violence and crime with mental illnesses (13). Although the overall likelihood of people with mental illnesses perpetrating violence is low, people tend to associate violence with mental illnesses because of inaccurate media portrayals. There is very little risk of violence or harm when a stranger encounters a person who has a mental illness (2). A 2005 study published in *Psychiatric Services* found that nearly 40% of all articles reporting on various aspects of mental health issues fell under the "dangerousness" category and emphasized violent crime, suicidal behavior, or self-injurious behavior (14).

The media's often skewed portrayal of people with mental illnesses can have devastating effects on the public. Surveys conducted in the 1950s and 1990s that explored the role stigma plays in the lives of people with mental illnesses found a prevalence rate of stigma in the 1990s that was twice as high as that in the 1950s (15). Researchers hypothesized that the rise of entertainment media was largely responsible for this increase. Additional research has indicated that through the various forms of media, people with mental illnesses are represented as dangerous 75% of the time (15).

By encouraging accurate and outstanding mental health journalism, the fellowship program enables six domestic journalists and four international journalists each year to produce a significant project on mental health or mental illnesses. Fellows are awarded $10,000 and make two expense-paid trips to The Carter Center to meet with other fellows, the Journalism Fellowship Advisory Board, and The Carter Center Mental Health Task Force. The Carter Center Mental Health Program provides journalists with access to accurate mental health information and a network that includes alumni fellows, professional contacts, and experts in the mental health field. The fellows are each paired with a mentor from the Journalism Fellowship Advisory Board to guide them through the fellowship process and the completion of their projects. All awarded journalists and Journalism Fellowship Advisory Board members are part of an electronic listserv, an online communication tool connecting former and current fellows with the Journalism Fellowship Advisory Board members and Mental Health Program staff. The listserv provides a forum through which fellows can educate one another about rising challenges in reporting on mental health, develop story ideas, and provide contacts and resources. An online archive of all previous and current fellows' work is available from www.cartercenter.org.

In September 2005, The Carter Center hosted the ninth class of fellows; the nine classes have included 68 fellows. Projects have included newspaper and magazine articles, national radio pieces, four published books, and five television documentaries. Previous topics and issues addressed include the delivery of mental health services to multiethnic populations, school programs, suicide, schizophrenia, and discrimination and abuse of people who have mental illnesses. Many fellowship projects have garnered recognition and awards including two Pulitzer Prize nominations and several Emmy nominations. The success of the fellowship program has been a driving force in the reduction of stigma and discrimination surrounding mental illnesses by changing the public's perception through an informed media committed to accurate, balanced reporting.

The fellowship program has been extremely successful and productive in reaching out to diverse groups of people to educate them about mental health issues. For example, Jim Marbrook, a 2004–2005 New Zealand-based fellow, recently completed *Awa Hikoi: The River Journey*, a documentary on healing rituals and recovery of mental illnesses in the Maori tradition. The documentary aired on national television in New Zealand. In a series of articles for *The Oregonian*, another 2004–2005 fellow, Michelle Roberts, exposed how some Oregon State Hospital caretakers sexually abused mentally ill children. As a direct result, the president of the U.S. Senate demanded immediate action to address the systemic shortcomings outlined in Roberts' reporting. At the opening of the new session, policy makers approved new laws that addressed the major problems exposed by Roberts. The nine fellowship classes have produced more than 400 pieces, and many continue to produce quality products about mental health issues after the completion of their fellowship.

## Conclusion

The enormous burden of mental illnesses and the looming public health crisis of mental health care have a detrimental effect on society, and many barriers keep individuals with mental illnesses from seeking, obtaining, and maintaining treatment. The Carter Center Mental Health Program supports the implementation of lasting policy change by convening groups of mental health professionals, from consumer and advocacy groups to top government officials, and collaborating with them in their efforts.

By educating the public about mental illnesses, The Carter Center Mental Health Program attacks the greatest barrier, stigma. The Rosalynn Carter Fellowships for Mental Health Journalism are developing a group of committed journalists dedicated to portraying an accurate and balanced view of mental health issues, to educate the public about the illnesses that effect so many. With changing public perceptions about mental illnesses, the public health crisis can finally be addressed.
